# Bibliometric analysis of hotspots in *Staphylococcus aureus* drug resistance research

**DOI:** 10.3389/fmicb.2025.1572400

**Published:** 2025-05-02

**Authors:** Jianguo Shi

**Affiliations:** Department of Clinical Laboratory, First Hospital of Shanxi Medical University, Taiyuan, Shanxi, China

**Keywords:** *Staphylococcus aureus*, drug resistance, bibliometrics, knowledge map, analysis

## Abstract

**Objective:**

Bibliometric analysis was used to visualize the current literature data on *Staphylococcus aureus* drug resistance.

**Methods:**

We used a bibliometrix package that utilizes R 4.4.1, all *S. aureus* drug resistance related research literature in the WOS core database, and data visualization and analysis of the retrieved research literature.

**Results:**

A total of 3,253 research articles on *Staphylococcus aureus* resistance were screened, and the number of publications related to *Staphylococcus aureus* resistance is increasing year by year. China ranks first in the number of publications, and the United States ranks first in terms of citations. Frontiers in microbiology has published the most articles on this research topic. Wang Y, Lee JH, and Simoes M have published the most articles, and Franci G has the most citations. The Egyptian Knowledge Bank (EKB), the Chinese Academy of Sciences, and Harvard University are the main research institutes. A total of 15,824 authors have contributed to the field, with the majority of authors coming from China, followed by South Korea and the United States, and most of them coming from the Chinese Academy of Science and Jilin University. The mechanism of drug resistance in *Staphylococcus aureus* was the most important research hotspot, with *Staphylococcus aureus* coming up 984 times, (10%), followed by antibiotic-resistance (693 times, 7%) and biofilm formation (517 times, 5%).

**Conclusion:**

This study comprehensively summarizes past research trends in *Staphylococcus aureus* resistance and identifies the countries, institutions, authors, journals, and publications involved in this field. The results provide a comprehensive overview of the study of resistance in *Staphylococcus aureus*.

## 1 Introduction

With the discovery of penicillin in the 20th century and its subsequent clinical application, humankind entered the era of antibiotics (Aminov, 2010). However, with the widespread use of penicillin, penicillin-resistant *Staphylococcus aureus* was soon discovered. Studies have shown that *Staphylococcus aureus* produces an enzyme that can hydrolyze penicillin (Kirby, 1944). In 1959, methicillin, a new semisynthetic penicillin that can resist penicillinase, was applied in the clinic to solve the problem of penicillin resistance (Palavecino, 2004). However, methicillin-resistant *Staphylococcus aureus* (MRSA) was identified in 1961 (Eriksen, 1961), and MRSA soon spread around the world (Mendes et al., 2014). The widespread use and even abuse of antibiotics has made bacterial resistance a difficult problem that people have to face. With the deepening of research, people have a more comprehensive understanding of the mechanism of bacterial resistance. The cause of methicillin resistance of *Staphylococcus aureus* is the presence and expression of the mecA gene.

The mecA gene is located on “staphylococcal cassette chromosome mec” (SCCmec), a mobile element in the chromosomal DNA of *Staphylococcus aureus*, and encodes penicillin-binding protein 2a (PBP2a). PBPs play an irreplaceable role in the process of cell wall synthesis, and *β*-lactam antibiotics can bind to PBPs to inactivate them, resulting in bacterial death. However, PBP2a binds to *β*-lactam antibiotics at a low rate, so PBP2a can participate in cell wall synthesis in place of other PBPs, resulting in resistance of *Staphylococcus aureus* to *β*-lactam antibiotics (Lim and Strynadka, 2002).

The origin of the SCCmec element is unknown, but it can integrate many exogenous resistance genes into it, leading to multidrug resistance in *Staphylococcus aureus* (Rolo et al., 2014). Therefore, MRSA exhibits greater pathogenicity and more extensive resistance than methicillin-susceptible *Staphylococcus aureus* (MSSA).

Vancomycin is the last line of defense for MRSA infection, but there have been cases of *in vitro* vancomycin sensitivity with insignificant or even failed treatment (Hiramatsu, 2001). Vancomycin-resistant *Staphylococcus aureus* (VRSA) was first reported in the United States in 2002 (Hiramatsu, 2001), and VRSA has been reported since then (Kumar, 2016). MRSA and other pan-resistant bacteria have been referred to as “superbugs” (Nordmann et al., 2007). In 2014, the World Health Organization (WHO) issued a report warning that the “post-antibiotic era” was coming (World Health Organization, 2014).

Many studies have been conducted to investigate the resistance of *Staphylococcus aureus* to various types of antibiotics including: *β*-lactam antibiotics, oxazolidinone antibiotics, aminoglycoside antibiotics, tetracycline antibiotics, and so on. However, due to the misuse of antibiotics, new resistant bacteria are still being produced. Drug resistance of *Staphylococcus aureus* (*S. aureus*) has always been a research hotspot in the related fields, and in the environment of explosive growth of various research papers, it is particularly important to summarize and analyze the huge amount of literature information by using scientific methods. Therefore, this study analyzed the literature on drug resistance of *Staphylococcus aureus* based on bibliometrics with the aim of comprehensively and objectively integrating and describing the current status of the research in this field and the future development trend, so as to provide a reference basis for subsequent related research.

## 2 Research information and methods

### 2.1 Sources of data

Literature related to *Staphylococcus aureus* drug resistance was obtained from the Science citation Index Expanded (SCI-expand) in the WOS core database: [TS = (“*Staphylococcus aureus*” OR “methicillin-resistant *staphylococcus aureus*” OR “s. aureus” OR “*staphylococcus aureus*”)] AND [TS = (“Antimicrobial resistance*” OR “Drug Resistance*” OR “Antibiotic Resistance” OR “Antimicrobial Drug Resistances*” OR “Drug Antimicrobial Resistance*” OR “antibiotic resistance genes” OR “drug-resistance”)] AND [TS = (“Biofilm*” OR “antibiofilm” OR “bacterial biofilm*”)] were the subject terms used to retrieve all the English literature. We set the publication date as December 20, 2024, set the type of literature were original research, i.e., “Article” or “Review Article,” and stored it in text format. The results of the search included the country, organization, author and publication date of all the literature, excluding duplicates and incomplete information.

### 2.2 Descriptive analysis

The bibliometrix (Aria and Cuccurullo, 2017) program package in R 4.4.1 was used to analyze the annual number of publications in the relevant literature as well as information on the number of relevant publications and the number of relevant citations for countries, institutions, authors, journals, and literature. Data analysis of high-frequency keywords was also performed using the bibliometrix package, and the visualization was presented as a word cloud and a tree diagram.

### 2.3 Collaborative network analysis and keyword co-occurrence analysis

The bibliometrix package in R4.4.1 was used to analyze the collaborative relationships between authors of related literature, plot the collaborative network, timeline, and density graph, and to analyze and plot the keyword co-occurrence network graph. The larger the number of related publications, the larger the nodes, the closer the nodes are connected, the wider the connecting lines, and the nodes displaying the same color indicate similarity in type or close cooperation.

## 3 Results

### 3.1 Annual global issuances

The search and screening in the WOS website yielded 3,323 relevant English-language articles without duplicates, and 70 irrelevant articles were excluded after reading the titles and abstracts. Finally, 3,253 English-language articles related to *Staphylococcus aureus* drug resistance were included for analysis.

**Figure 1:**
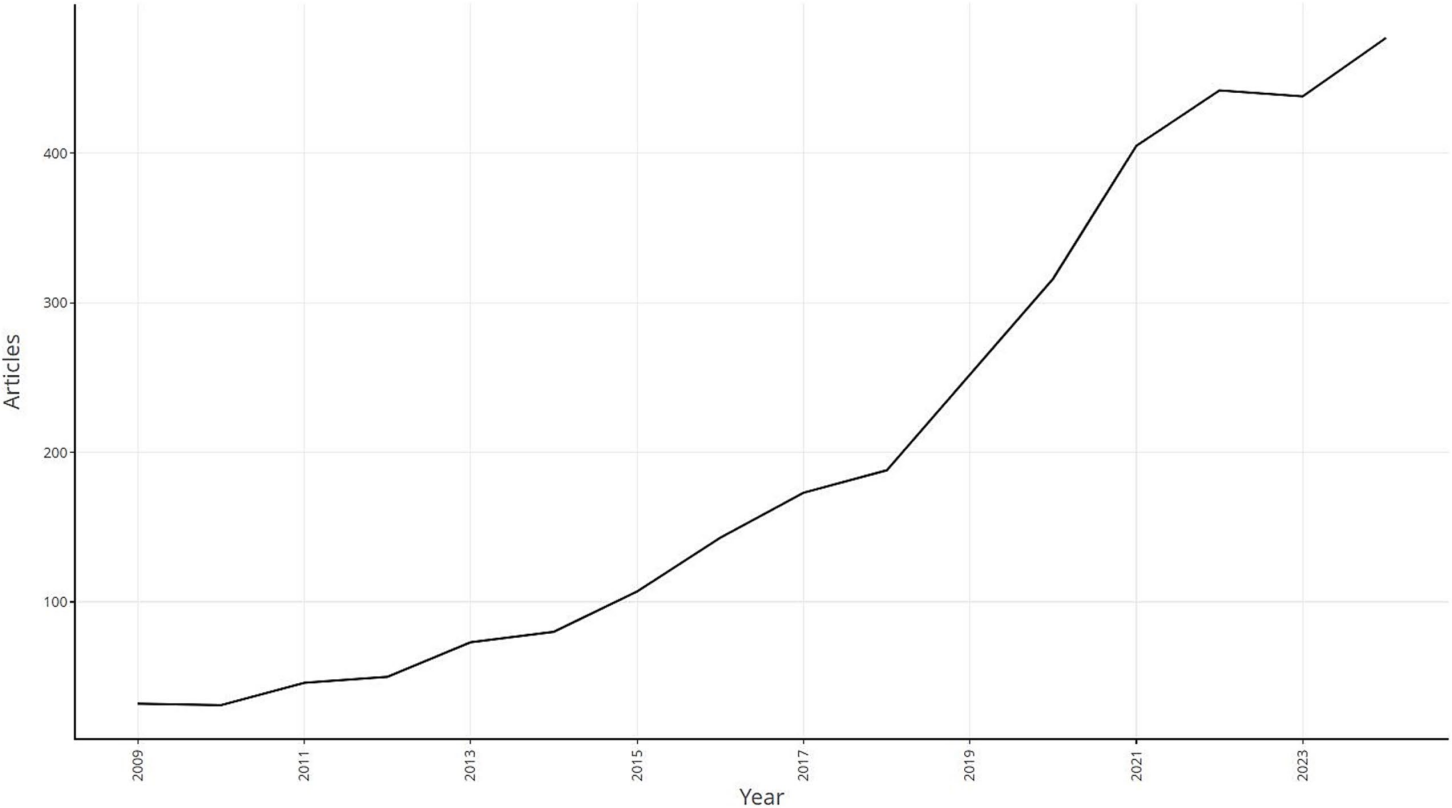
Publication of related literature.

Web of science studies on *S. aureus* drug resistance were first published in 2009, and in general the number of relevant publications has been on an upward trend since its inception. The number of relevant articles peaked at 477 in 2024, which has great research value and development potential (Figure 1).

### 3.2 National (regional) publications and citations

**Figure 2:**
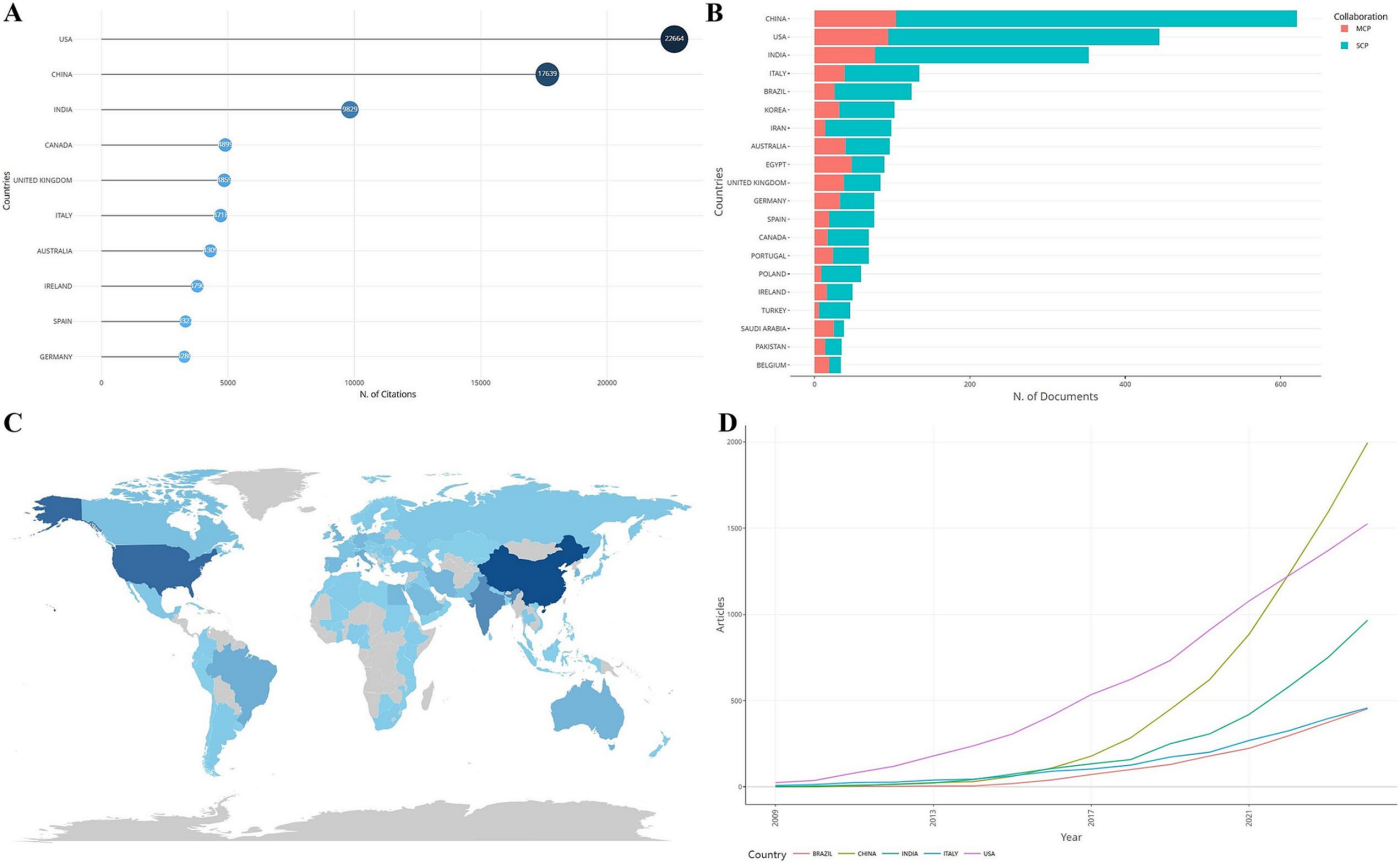
Issuance and citations by country. **(A)** Top 10 countries in terms of citations to relevant literature. **(B)** SCP and MCP issuance. **(C)** Distribution of country issuance maps. **(D)** Cumulative growth of articles over time.

Statistics on the number of *S. aureus* drug resistance-related publications, the number of single country publications (SCPs), the number of multiple country publications (MCPs), and the number of citations in the countries (regions) to which the corresponding authors belonged showed that a total of 84 countries (regions) contributed to this field of research (see Figure 2 for a map of the countries’ publications, with darker colors showing higher number of publications). Table 1 shows the top ten countries in terms of the number of relevant publications, among which China ranked first with 621 relevant publications, the United States ranked second with 444 relevant publications, and India ranked third with 353 relevant publications. Figure 2A shows the top 10 countries in terms of citations, with Italy ranking first (479 citations) and China ranking third (307 citations). The results of the change over time in the number of articles issued by each of the top five countries show that at the beginning, there was a difference between the number of articles issued by each country and the rate of growth of the number of articles issued, but not much, and then the United States gradually increased the rate of growth of the number of articles issued and opened up a certain gap with the other countries. The growth rate of China’s publications increased sharply from 2017 onwards, and by 2022, China’s publications far exceeded those of other countries (see Figure 2). China ranks first in terms of the number of relevant publications and second in terms of citations, indicating that China’s international recognition and influence in this research field still needs to be improved. The top countries in terms of the number of relevant publications, such as China, the United States, India, etc., published mostly SCP. The percentage of MCP shows that there are more MCPs in this field, with the highest percentage of MCPs in EGYPT, which indicates that there are more collaborations between the countries and other countries (Figure 2B; Table 1).

**Table 1:** Top 10 countries in terms of relevant publications.

Ranking	Country	Literatures	SCP	MCP	MCP ratio (%)
1	CHINA	621	516	105	16.9
2	USA	444	349	95	21.4
3	INDIA	353	275	78	22.1
4	ITALY	135	96	39	28.9
5	BRAZIL	125	99	26	20.8
6	KOREA	103	71	32	31.1
7	IRAN	99	85	14	14.1
8	AUSTRALIA	97	57	40	41.2
9	EGYPT	90	42	48	53.3
10	UK	85	47	38	44.7

### 3.3 Journal publications and citations

**Figure 3:**
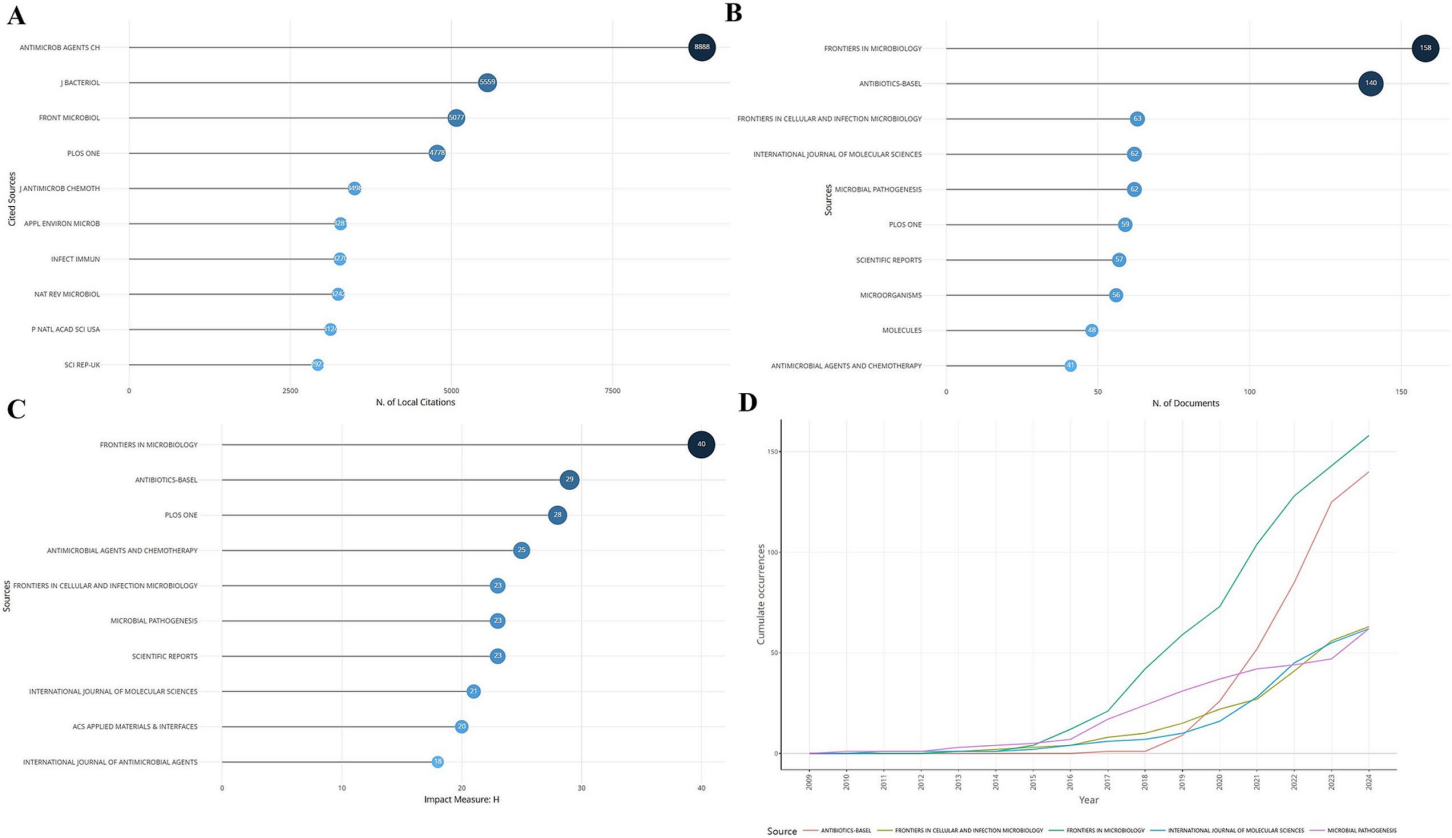
Journal publications and citations. **(A)** Ranking of local citations by journal. **(B)** Ranking of publications by journal. **(C)** Ranking of impact by journal. **(D)** Cumulative publications by journal over time.

Statistics of journals that published literature related to *S. aureus* drug resistance showed that the top 10 journals in terms of local citations are shown in Figure 3A, with Antimicrob Agents CH being the first with 8,888 local citations. Analysis of the journals most relevant to *S. aureus* drug resistance showed that Frontiers in Microbiology published the largest number of articles related to the topic of this study, with 158 articles from this journal (Figure 3). The impact ranking of journals showed that Frontiers in Microbiology had the highest impact. The analysis of the cumulative number of articles published in the relevant literature of the top five journals showed that from 2009 to 2015, the cumulative number of articles published in each journal has gradually increased, and the number of articles published in Frontiers in Microbiology had ridden high after 2015 and was much higher than other journals by 2024.

### 3.4 Article citations

**Figure 4:**
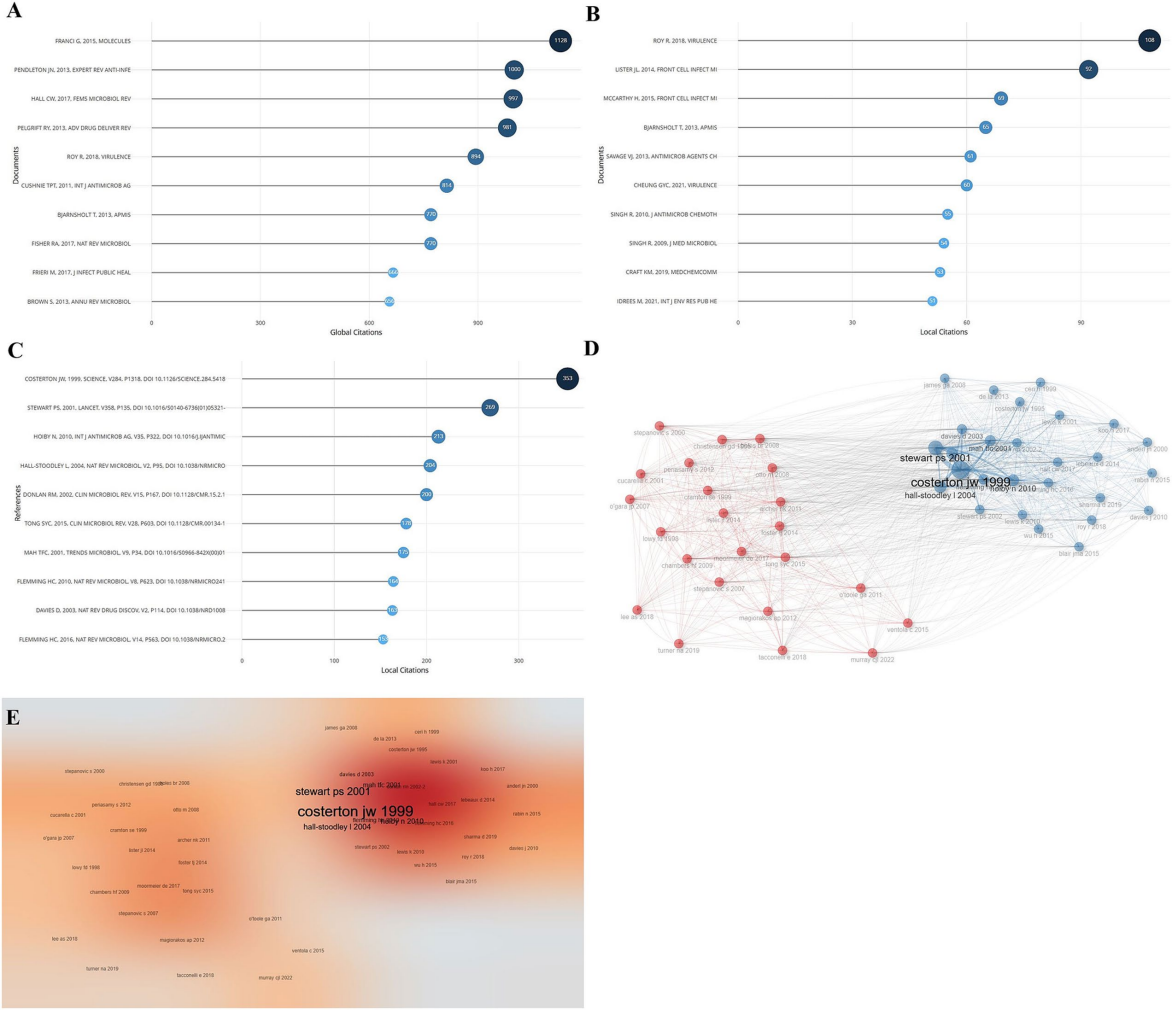
Citation of articles. **(A)** Ranking of the most cited articles globally. **(B)** Ranking of the most cited articles locally. **(C)** Ranking of the most cited literature in each article. **(D)** Cluster analysis of citation networks. **(E)** Citation network hotspot map.

The citations of published literature related to *Staphylococcus aureus* drug resistance were counted, and the top 10 articles in terms of relevant citations are shown in Figure 4. Among them, the top 3 articles in the global citation rankings were from authors Franci G (1128), Pendleton JN (1000), and Hall CW (997) (Figure 4A); the top 3 articles in the local citation rankings were from authors Roy R (108), Lister JL (92), and McCarthy H (69 times), see Figure 4B. The most cited article in the references of the articles included in this analysis was the article published by Costerton JW et al. in Science (353 citations), see Figure 4C. The results of the citation co-occurrence network of the top-ranked articles show that they are divided into two main categories, see Figure 4D, and the heat map of the citation co-occurrence network is shown in Figure 4E.

### 3.5 Institutional issuance

**Figure 5:**
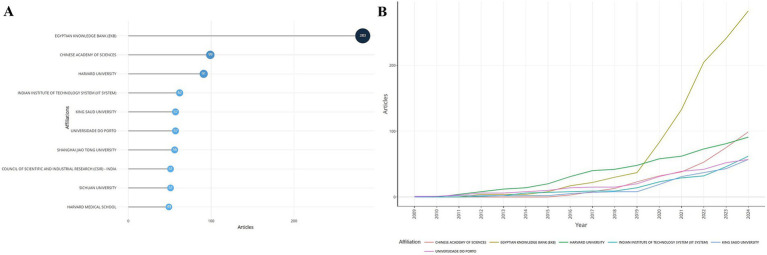
Institutional issuances. **(A)** Ranking of institutions in terms of the number of issuances. **(B)** Changes in the number of issuances by institution over time.

A total of 2,645 institutions contributed to the field. Among them, the top 10 institutions in terms of the number of articles published are shown in Figure 5A, and the top 3 were the Egyptian Knowledge Bank (EKB), the Chinese Academy of Sciences, and Harvard University. The trend of the number of articles issued by each organization over time is shown in Figure 5, which demonstrates a steady upward trend for all organizations, with the number of articles issued by the EKB rising sharply after 2019 to 2024, when it had far surpassed that of other organizations.

### 3.6 Author’s publications

**Figure 6:**
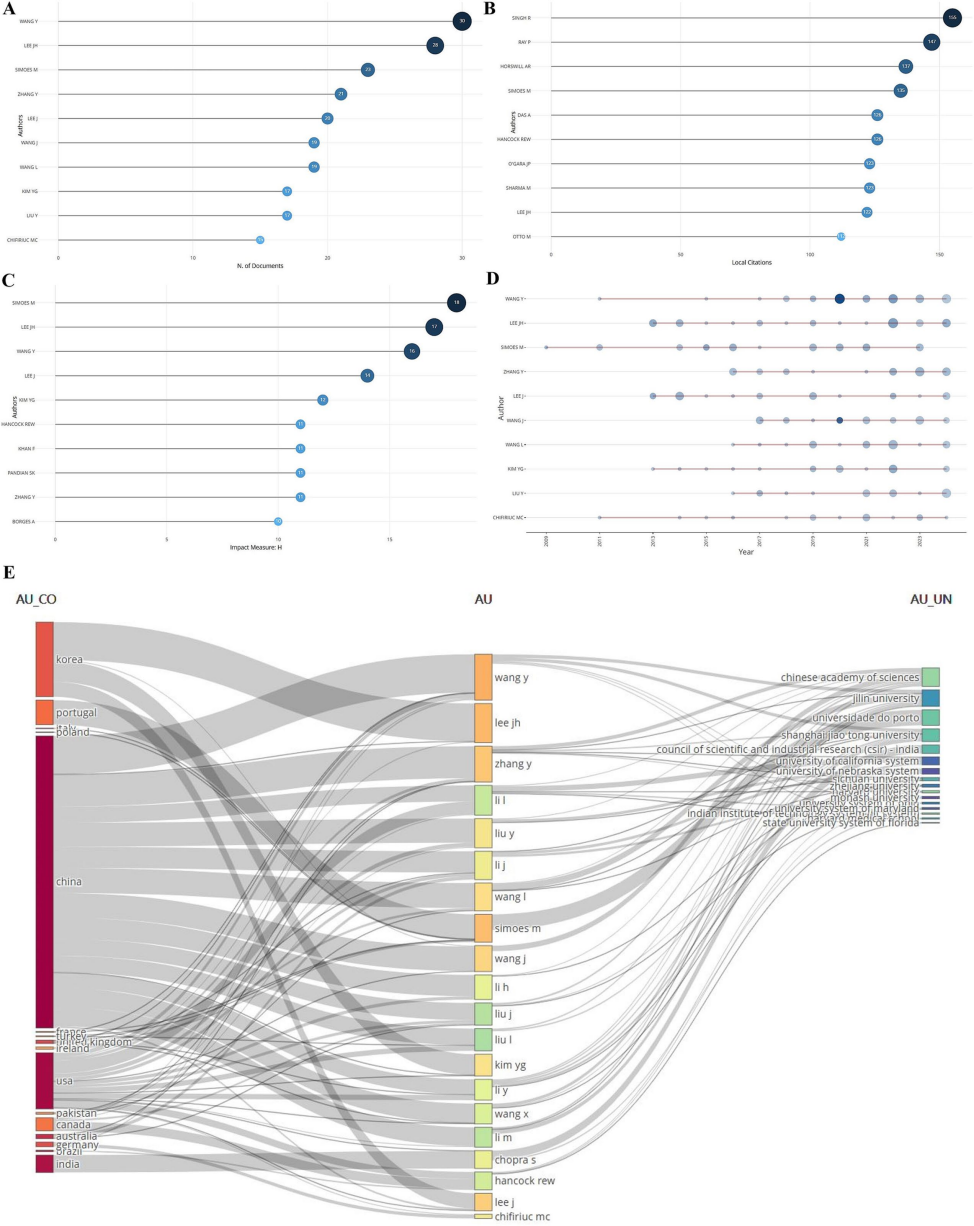
Authors’ publications. **(A)** Ranking of number of publications. **(B)** Ranking of local citations. **(C)** Ranking of impact. **(D)** Changes in authors’ publications over time. **(E)** Authors’ countries and keywords used in published articles (country names on the left, authors’ names in the center, and institutions’ names on the right).

A total of 15,824 authors had contributed to the field. Among them, the top 10 authors in terms of the number of publications are shown in Figure 6A, and the top three are Wang Y (30), Lee JH (28), and Simoes M (23); in the ranking of the number of local citations, Singh R was ranked No. 1 with 155 citation frequency (Figure 6B); and in the ranking of the authors’ influence, it was Simoes M was ranked No. 1 (Figure 6C); and the authors’ publication status showed that each of the top 10 authors in this field from 2013 to 2024 have published a lot of articles one after another (Figure 6D); Figure 6E showed that most of the authors are from China, followed by South Korea and the U.S.A., and most of these authors were from the Chinese Academy of Sciences and Jilin University.

### 3.7 Analysis of cooperation networks

**Figure 7:**
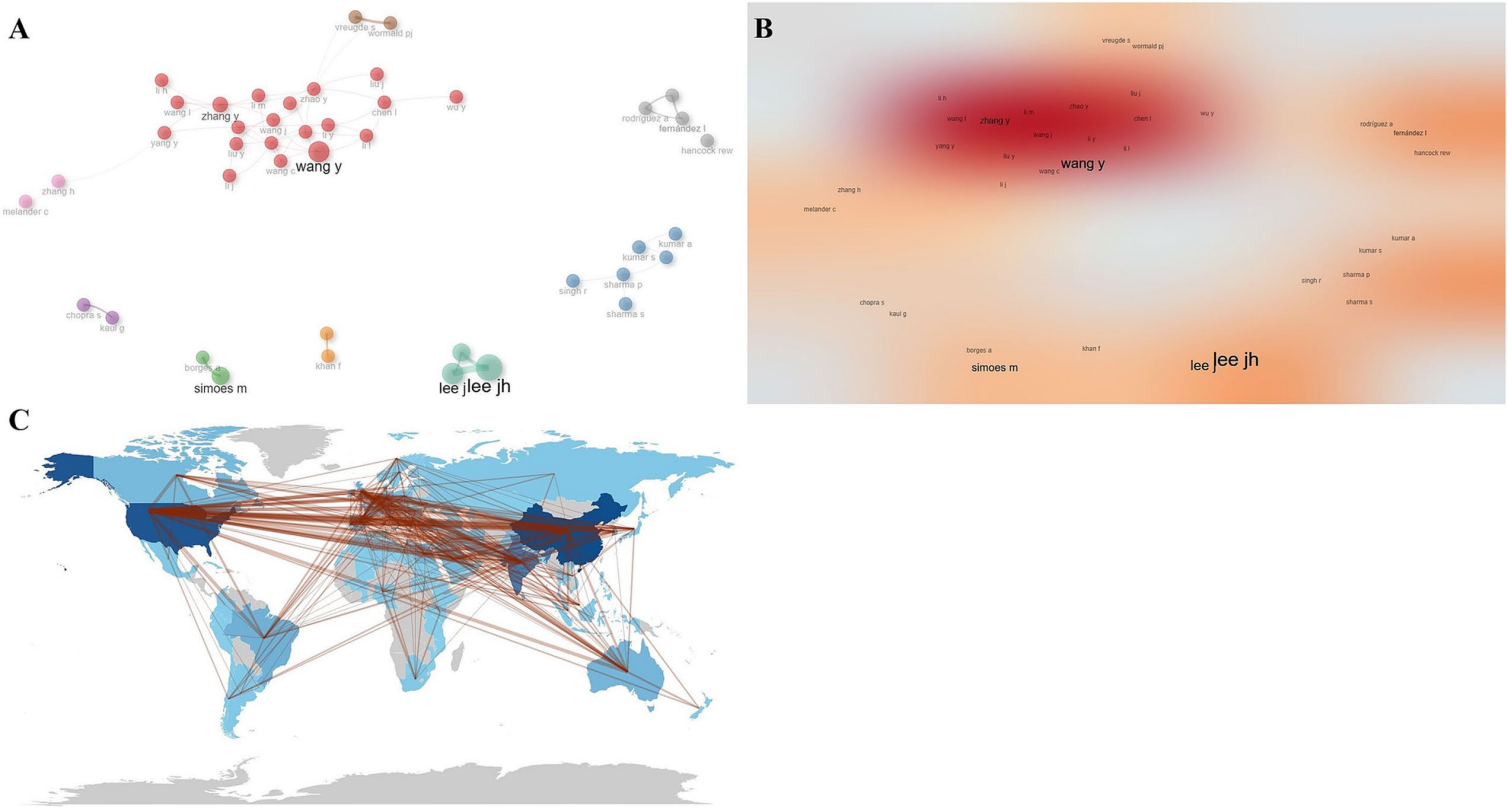
Collaborative network analysis. **(A)** Author collaborative network map. **(B)** Author collaborative network heat map. **(C)** Country collaborative network map.

Collaborative network analysis of the included authors showed that there was a total of nine collaborative groups, with the teams of Wang Y et al. and Zhang Y et al. collaborating most closely, and the teams collaborating more independently and less with each other (Figures 7A,B). The country cooperation graph showed that the countries that cooperated most closely with other countries were mainly China and the United States (Figure 7C).

### 3.8 Analysis of high-frequency keywords and keyword co-occurrence networks

**Figure 8:**
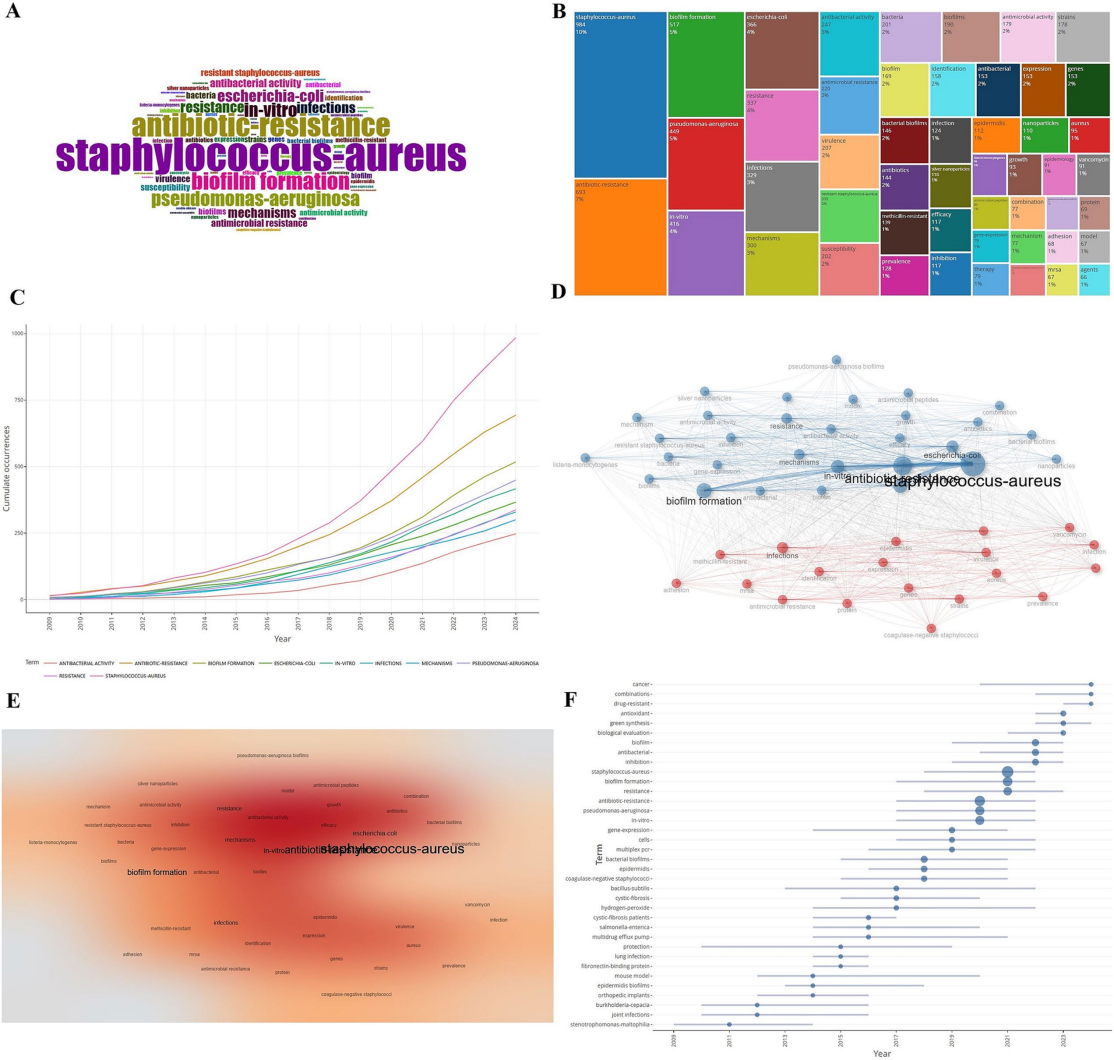
Analysis of high-frequency keywords and theme changes. **(A)** Word cloud map. **(B)** Keyword tree map. **(C)** Keyword frequency over time. **(D)** Keyword co-occurrence network. **(E)** Keyword co-occurrence heat map. **(F)** Theme evolution map.

Keywords are extractions of key informations from a piece of literature and the core and hotspot of the research field can be roughly inferred by understanding the high-frequency keywords appearing in the related research. The high-frequency keywords appearing in the literature related to drug resistance of *Staphylococcus aureus* were statistically analyzed and a word cloud (Figure 8A) and a tree diagram (Figure 8B) were drawn: the larger area occupied by the keywords indicated the higher frequency of occurrence. As shown in Figure 8C the top three high-frequency words were *Staphylococcus aureus* (984 times, 10%) followed by antibiotic-resistance (693 times, 7%) and biofilm formation (517 times, 5%). The change in keyword frequency over time showed that the frequency level of each keyword increased over time with *Staphylococcus aureus* and antibiotic-resistance growing at a much higher rate than the other keywords. The 5,670 keywords appearing in the literature related to *Staphylococcus aureus* drug resistance will be analyzed for co-occurrence and the clustering of the top 50 keywords in the co-occurrence network is shown in Figures 8D,E which are mainly divided into two main categories. Cluster 1 is dominated by *Staphylococcus aureus* and antibiotic-resistance etc.; Cluster 2 is dominated by infections etc. The results of the theme evolution analysis show that a series of changes have occurred in the theme terms of this field from 2009 to 2024 from the beginning of pathogenic bacterial infections to drug resistance detection and gene expression to the exploration of drug resistance mechanisms (Figure 8F).

## 4 Discussion

*Staphylococcus aureus* is a common pathogen that occurs worldwide. Its resistance is mainly the result of overuse of antibiotics, and it is usually resistant to antibiotics such as beta-lactams. Methicillin-resistant strains (MRSA) first appeared in 1960 and were characterized by resistance to all *β*-lactam antibiotics used for treatment at that time. MRSA strains continued to spread from 1970–1980, when cephalosporins were used on a large scale in hospitals, but resistant strains soon appeared (Antimicrobial Resistance Collaborators, 2022; Wan et al., 2020; Feng et al., 2022). MRSA infections in hospitals become hospital-acquired MRSA (HA-MRSA) and infections in ambulatory patients developed into community-acquired CA-MRSA (Mlynarczyk-Bonikowska et al., 2022; Buommino et al., 2021). MRSA strain infections have always been a challenge for clinical treatment, and vancomycin has become the most effective drug for the treatment of MRSA; however, with the extensive use of vancomycin, strains that are moderately resistant to vancomycin have also been reported. β-lactam antibiotics and vancomycin are both bactericidal by interfering with the synthesis of the cell wall of *Staphylococcus aureus* (Mlynarczyk et al., 2009). Resistance to β-lactam antibiotics in MRSA strains is associated with the presence of metastable genomic elements in the bacterial genome, of which the mec gene determines resistance to methicillin. The genome evolved rapidly and slowly started to become resistant to antibiotics such as β-lactams, macrolides, tetracyclines, and aminoglycosides (Zhao et al., 2021; Ngoubangoye et al., 2023).

In this study, the related literature postings showed that the number of *S. aureus* drug resistance-related postings had been in an upward phase, in which the growth rate of postings increased further after 2018, which may be related to the inclusion of ESKAPE-resistant bacteria in the list of urgently needed new antibiotics for research and development by WHO in 2017 (De Oliveira et al., 2020). In this list, carbapenem-resistant *Acinetobacter baumannii* (CRAB), carbapenem-resistant *Pseudomonas aeruginosa* (CRPA), carbapenem-resistant *Klebsiella pneumoniae* (CRKP), and carbapenem-resistant Enterobacteriaceae (CRE) are included in the priority level, and vancomycin-resistant *Enterococcus faecalis* (VRE) and methicillin-resistant *Staphylococcus aureus* (MRSA) are in the high priority level. Chinese scholars are ranked No. 1 in terms of the number of publications, and the number of publications already surpassed that of the United States by 2024, however, the number of citations is ranked No. 2 and there is a big gap to the No 1., the United States. In addition, most of the top 10 core journals are from Europe and the United States, with no Chinese journals on the list. The top three research institutions in terms of publications are the EKB, the Chinese Academy of Sciences, and Harvard University, and most authors come from China. Most of the authors are from the Chinese Academy of Sciences and Jilin University. Combined with the results of Figure 2D, it can be seen that the number of publications in the United States was much higher than that in China in the initial period, which may be because the United States has more resources to invest in the study of *Staphylococcus aureus* resistance. China’s economy has also developed rapidly in recent years, so the speed of publication of its articles is also accelerating, and it might finally surpass that of the United States. However, China is still relatively late in this field, so it needs to further improve its influence. It can be seen that overall the United States is in the leading position in this research field, but as domestic research scholars continue to deepen their research in the field, China’s literature issuance speed dramatically accelerated globally (Wang et al., 2021), and the research results of Chinese scholars gradually play an increasingly important role in the international academic arena.

In this study, through keyword co-occurrence clustering analysis, we found that the current research hotspots of drug resistance in *Staphylococcus aureus* mainly focus on drug resistance, biofilm formation, and infection. Biofilm is an extracellular composite structure, formed by a population of microorganisms attached to the surface of a matrix, which is surrounded by a highly hydrated extracellular polymer matrix produced by the bacteria themselves, and these matrices can act as a kind of protective shell enclosing the bacteria inside, which is a way of survival for the bacteria to adapt to the environment (Craft et al., 2019). It allows the bacteria to resist the host’s immune response and evade killing by antibiotics, and is the main cause of persistent bacterial infections, as well as MRSA antibiotic resistance and its prevalence (Cascioferro et al., 2021). MRSA biofilm resistance mainly involves the following aspects: (1) the presence of polymerizable mucopolysaccharides on the surface of the biofilm makes the entry of antibiotics into the cells limited; (2) the presence of metabolically inactive cells-persistent cells in the deepest layers, which are intrinsically resistant to antibiotics; and (3) the accumulation of the number of bacterial cells within the biofilm promotes horizontal inheritance of the resistance genes and the transfer.

The mechanisms of drug resistance in *Staphylococcus aureus* mainly include the following: (1) antibiotic inactivation (such as *β*-lactam antibiotics); (2) antibiotic efflux (such as tetracyclines, fluoroquinolone antibiotics); and (3) ribosomal protection (e.g., aminoglycosides, tetracyclines, macrolides, glycopeptide antibiotics, and β-lactams) (Vestergaard et al., 2019; Pence et al., 2015). In the face of the increasing trend of *S. aureus* drug-resistant bacterial infections and the dilemma of druglessness, new non-antibiotic therapeutic options have to be urgently sought in a whole-health framework, and phage therapy is one of the options (Kortright et al., 2019). Since phages are widely present in the internal and external ecosystems of the human body and have the property of specifically lysing bacteria, it is believed that phage therapy is expected to be an effective complement to AMR infections in the “post-antibiotic” era (Zhong et al., 2022). Nowadays, phages and their products have been playing an important role in the treatment of ESKAPE-resistant bacterial infections, and several foreign case reports have shown that phage cocktail preparations have good clinical value for VRE infections and MRSA infections (Paul et al., 2021).

Currently, there are still many articles published on *S. aureus* drug resistance every year, and the time of publication of the literature can somehow reflect the academic research process and the speed of development in this field. Based on the results of this study, it is reasonable to believe that the research on *S. aureus* drug resistance will continue and be dynamic, so this study has further significance and value. In future research, we first need to integrate the literature of multiple databases to make the screening data as comprehensive as possible, and actively communicate with scholars in related fields to understand the cutting-edge developments in the field, to enhance and deepen the objective knowledge of the field, and to avoid personal subjectivity in the analysis and interpretation as much as possible.

Of course, this study also has some limitations. First of all, bibliometric analysis has high norms and standards for data, so in order to ensure the quality and completeness of the collected data, this study only selected journal articles in the SCI index of the core collection of the Web of science database and excluded other databases (e.g., Scopus), which inevitably leads to the problem of analyzing data that are not comprehensive enough. In addition, quantitative analysis needs to analyze and interpret the data, which requires the researcher to have a more in-depth and comprehensive understanding of the field, otherwise there will inevitably be a certain degree of subjectivity.

## 5 Conclusion

To summarize, the research related to *Staphylococcus aureus* drug resistance was first published in 2009 and the research fever is increasing year by year. The most authoritative author in the field of research on *Staphylococcus aureus* drug resistance is Simoes M. Frontiers in Microbiology is showing increased interest in the field of research on *Staphylococcus aureus* drug resistance.

## Data Availability

The original contributions presented in the study are included in the article/supplementary material, further inquiries can be directed to the corresponding author.
